# Chemical Hemisynthesis of Sulfated Cyanidin-3-*O*-Glucoside and Cyanidin Metabolites

**DOI:** 10.3390/molecules26082146

**Published:** 2021-04-08

**Authors:** Sarah Straßmann, Maike Passon, Andreas Schieber

**Affiliations:** Department of Nutritional and Food Sciences, Molecular Food Technology, University of Bonn, Friedrich-Hirzebruch-Allee 7, D-53115 Bonn, Germany; sarah.strassmann@magenta.de (S.S.); schieber@uni-bonn.de (A.S.)

**Keywords:** anthocyanins, cyanidin-3-*O*-glucoside, cyanidin, LC-IMS, HRMS, sulfation, metabolites

## Abstract

The metabolism of anthocyanins in humans is still not fully understood, which is partly due to the lack of reference compounds. It is known that sulfation is one way of the complex phase II biotransformation mechanism. Therefore, cyanidin-3-*O*-glucoside and the cyanidin aglycone were chemically converted to their sulfates by reaction with sulfur trioxide-N-triethylamine complex in dimethylformamide. The reaction products were characterized by UHPLC coupled to linear ion trap and IMS-QTOF mass spectrometry. Based on MS data, retention times, and UV-Vis spectra, the compounds could tentatively be assigned to A-, C-, or B-ring sulfates. Analysis of urine samples from two volunteers after ingestion of commercial blackberry nectar demonstrated the presence of two sulfated derivatives of the cyanidin aglycone and one sulfated derivative of the cyanidin-3-*O*-glucoside. It was found that both the A ring and the B ring are sulfated by human enzymes. This study marks an important step toward a better understanding of anthocyanin metabolism.

## 1. Introduction

Anthocyanins are water-soluble flavonoids that are widely found in the plant kingdom, for example, in berry fruits. Whereas the profile of some berries such as blueberries is rather complex, other fruits like strawberries and blackberries show a relatively simple composition, with pelargonidin-3-*O*-glucoside and cyanidin-3-*O*-glucoside respectively being the predominant compounds [1].

While it is well known that anthocyanins are poorly bioavailable, a large body of evidence suggests that they impart health benefits. Because anthocyanins have been demonstrated to be extensively metabolized, it is quite obvious that the metabolites rather than the parent compounds constitute the bioactive agents [2]. Most research on anthocyanin metabolites has focused on low-molecular phenolic compounds that result from the degradation of the flavonoid aglycone. However, metabolism of the intact anthocyanins and the aglycone may also occur, for example, by sulfation, glucuronidation, and methylation. In contrast to studies on the latter two pathways, the sulfation of anthocyanins has less frequently been reported, which may be due to the fact that sulfated metabolites are formed less often [3], the lack of reference compounds, and difficulties in sample preparation. However, the availability of standard substances is a prerequisite for the elucidation of the pharmacokinetics of anthocyanins.

Significant progress has been made in the synthesis of metabolites by enzymatic routes. For example, sulfated derivatives of cyanidin and cyanidin-3-*O*-glucoside were obtained employing porcine liver enzymes [4] and may be used for reference in databases. Because the yields were poor and enzymatic hemisynthesis is expensive, alternate approaches are needed.

Chemical hemisynthesis may be a more suitable tool to generate sulfated metabolites of anthocyanins. However, the sensitivity of anthocyanins to light, pH changes, and high temperature poses a problem [5], as does the lability of the sulfate groups under acidic conditions and at high temperatures [6]. As a result of these stability issues, synthesis should be carried out with as few steps as possible and under mild conditions. In anthocyanins, two types of hydroxyl groups are present, which are the aromatic OH groups of the anthocyanidin aglycone and the aliphatic OH groups of the sugar moiety. Because to our knowledge, there is no report about sulfations in the human body taking place on the sugar hydroxyl groups of anthocyanins, we aimed at the chemical hemisynthesis of anthocyanins sulfated on the flavonoid moiety. For this purpose, we used cyanidin-3-*O*-glucoside obtained from blackberry juice and the cyanidin aglycone prepared according to a previously described protocol [4]. Subsequently, we conducted a pilot study with two participants, who consumed blackberry juice and provided urine samples for analysis by LC-MS. This, in turn, was compared to the synthesized reference substances. Among other phase II metabolites of cyanidin, sulfated cyanidin-3-*O*-glucoside and sulfated cyanidin were detected. The aim of this work was to generate authentic reference substances for the identification of sulfated metabolites in real human biological samples.

## 2. Results and Discussion

**Scheme 1:**
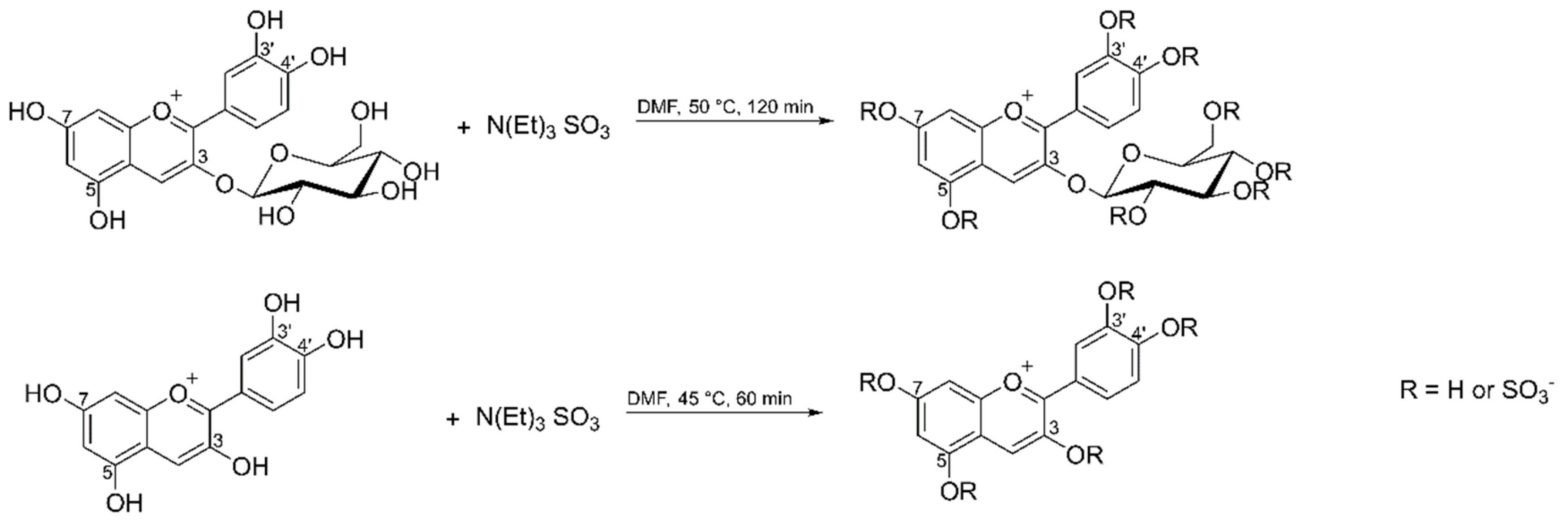
Synthesis reactions of cyanidin sulfates and cyanidin-3-*O*-glucoside sulfates.

The present study aimed at the chemical hemisynthesis of sulfated cyanidin-3-*O*-glucoside and cyanidin. For this purpose, the anthocyanin was purified from blackberry juice because blackberries have a relatively simple profile of pigments, cyanidin-3-*O*-glucoside being the predominant compound. Subsequently, the aglycone cyanidin was released by acidic hydrolysis. Both compounds reacted with a sulfur trioxide-*N*-triethylamine complex to yield a mixture of different mono- and disulfates (Scheme 1).

### 2.1. Cyanidin Sulfates

**Figure 1:**
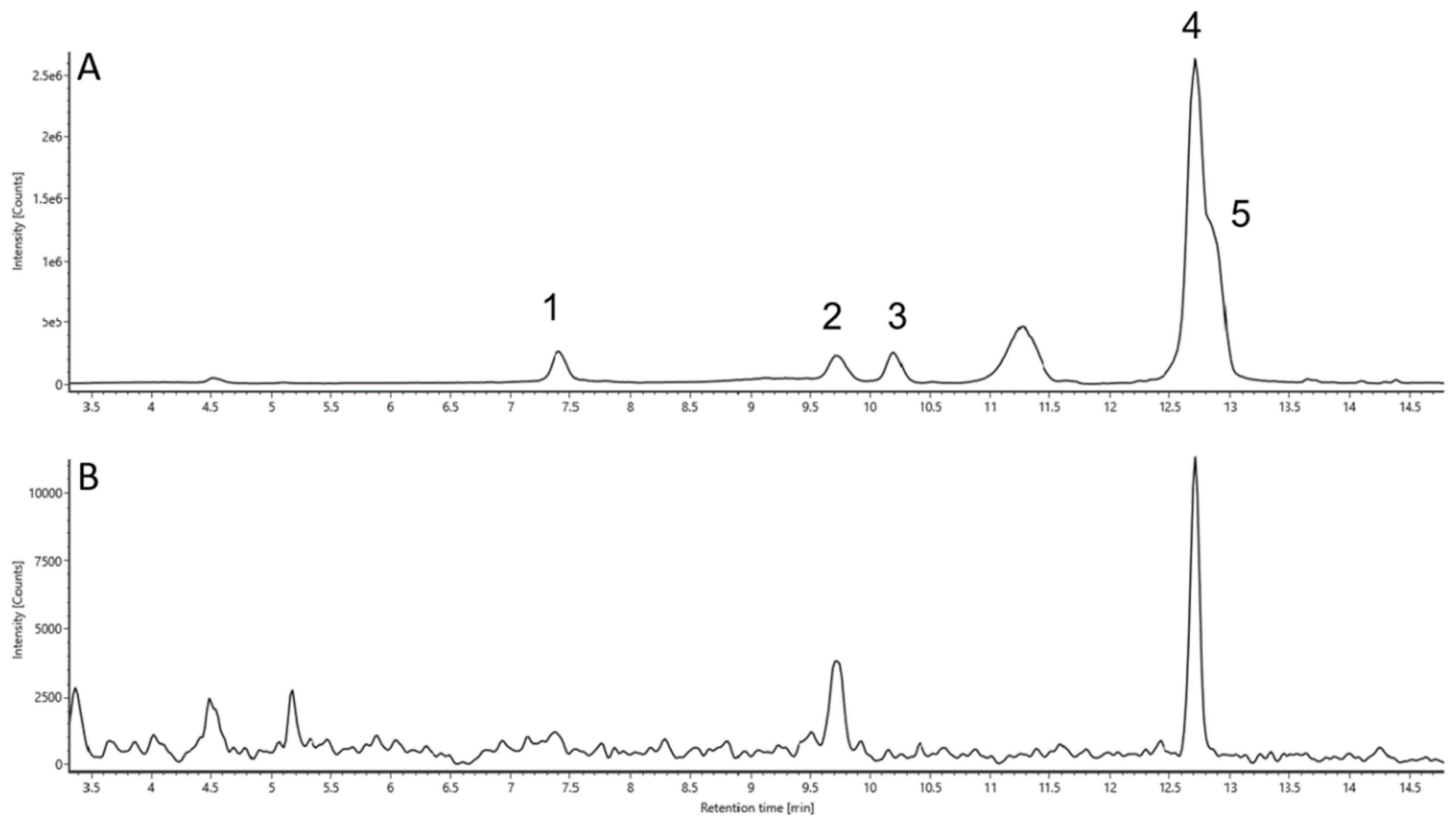
Extracted ion chromatograms of the [M]^+^, *m*/*z* 367.0118 (cyanidin monosulfate) after the synthesis reaction (**A**) and of the 24 h urine sample (**B**).

The extracted ion chromatogram of the [M]^+^ of monosulfated cyanidin is shown in Figure 1A. Five peaks belonging to monosulfates (**1**–**5**) and at least two peaks (RT 4.5 and 11.3 min) which are in-source fragments of disulfates can be observed. While absolute quantification was not possible due to the lack of references, the relative distribution of the monosulfates could be determined. Thus, compound **1** was formed at 4%, compound **2** at 1%, compound **3** at 3%, compound **4** at 50%, and compound **5** at 42%.

Identification was based on fragmentation patterns, accurate masses, CCS (collision cross-section) values, chromatographic behavior, and UV-spectra (Table 1). The interpretation of these data allows a tentative assignment of the site of sulfation to the A, B or C ring, which is described as follows.

#### 2.1.1. Retention Time

The retention time may be indicative of the site of sulfation and should be taken into consideration for tentative peak assignment. From Figure 1A it can be seen that peak **1** elutes very early and remote from all other cyanidin sulfates. The chromatographic behavior of peaks **4** and **5** is very similar, resulting in partial coelution, which suggests a structural similarity. According to previous studies, quercetin sulfated at the 3-OH position elutes earlier than derivatives that bear sulfate groups on the 3′- or 4′-OH positions [7,8]. The elution order found for quercetin monosulfates was 3, 5, 7, 4′, and 3′ [9]. Taking into account the structural similarity of flavonoids and the identical substitution pattern of the B ring of quercetin and cyanidin, it is reasonable to assume this elution order also for cyanidin monosulfaltes.

#### 2.1.2. UV/Vis Spectra

**Figure 2:**
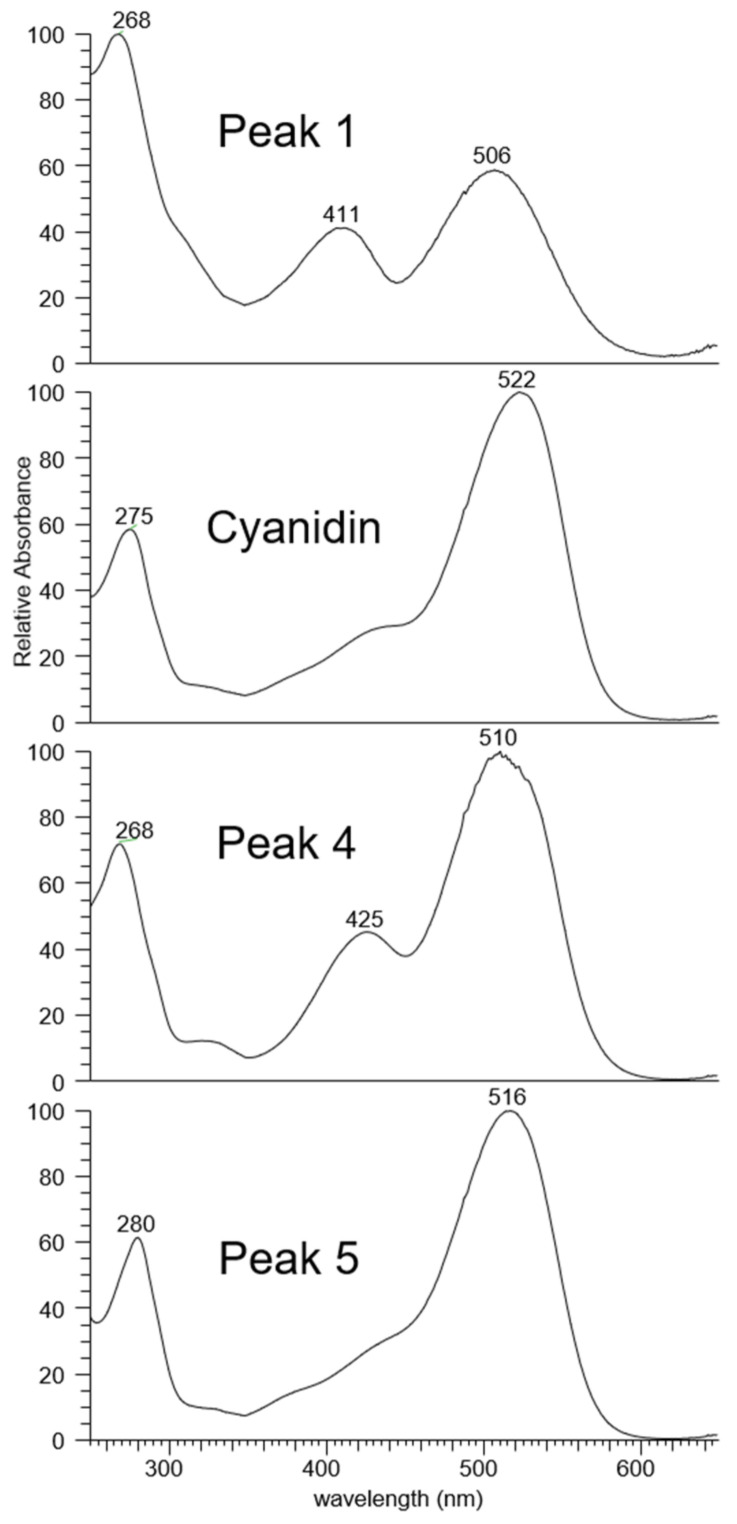
UV/Vis spectra of cyanidin (RT 11.7 min) in comparison to three cyanidin monosulfates (RT 7.1, 12.6 and, 12.8 min).

Consideration of the shifts of the λ_max_ is another useful way of identifying the individual compounds formed in this reaction. Anthocyanins show a distinctive band I in the 450–560 nm region and band II in the 240–280 nm region [10,11]. Sulfation of cyanidin led to a hypsochromic shift in the spectrum of all peaks **1**–**5**. Peak **1** showed the largest shift in comparison to cyanidin in band I from 525 nm to 505 nm and in band II from 277 to 267 nm. Shifts in the range of 2 to 13 nm were observed for all other peaks. A comparison of the UV/Vis spectra of cyanidin, peak **1** and the two most abundant monosulfated cyanidin derivatives (peaks **4** and **5**) is shown in Figure 2. Hypsochromic shifts of the absorption maxima of quercetin through sulfation are already reported in the literature [9]. Here, the 3-OH sulfation caused also the highest shift of 21 nm in band I, whereas conjugation at the 5-, 4′-, and 3′-OH resulted in a small shift; sulfation of the 7-OH position did not affect the λ_max_. Interestingly, the formation of a new band at approximately 420 nm was observed, which constitutes an even bigger difference between the sulfated cyanidin molecules than the shift of the maxima. Peaks **1** and **4** show this band at 411 nm and 425 nm respectively, whereas sulfation at the other positions either does not affect this band, or the compound concentrations were too low. However, it is possible to determine the ratio of the absorption at 440 nm and the absorption maximum (λ_440nm_/λ_max_). This ratio is 70% for peaks **1**–**3**, 50% for peak **4** and 30% for peak **5**, cyanidin, and cyanidin-3-*O*-glucoside. This ratio is already described in the literature [12], where it is reported that the ratio of an anthocyanin 3-*O*-glucoside is twice as high as that of a 5-*O*-glucoside. Larger shifts are caused by the disulfates, as can be seen from Table 1. Due to the high number of possible combinations and low substance concentrations, no conclusion concerning the conjugation positions can be drawn.

#### 2.1.3. Mass Spectrometry

**Figure 3:**
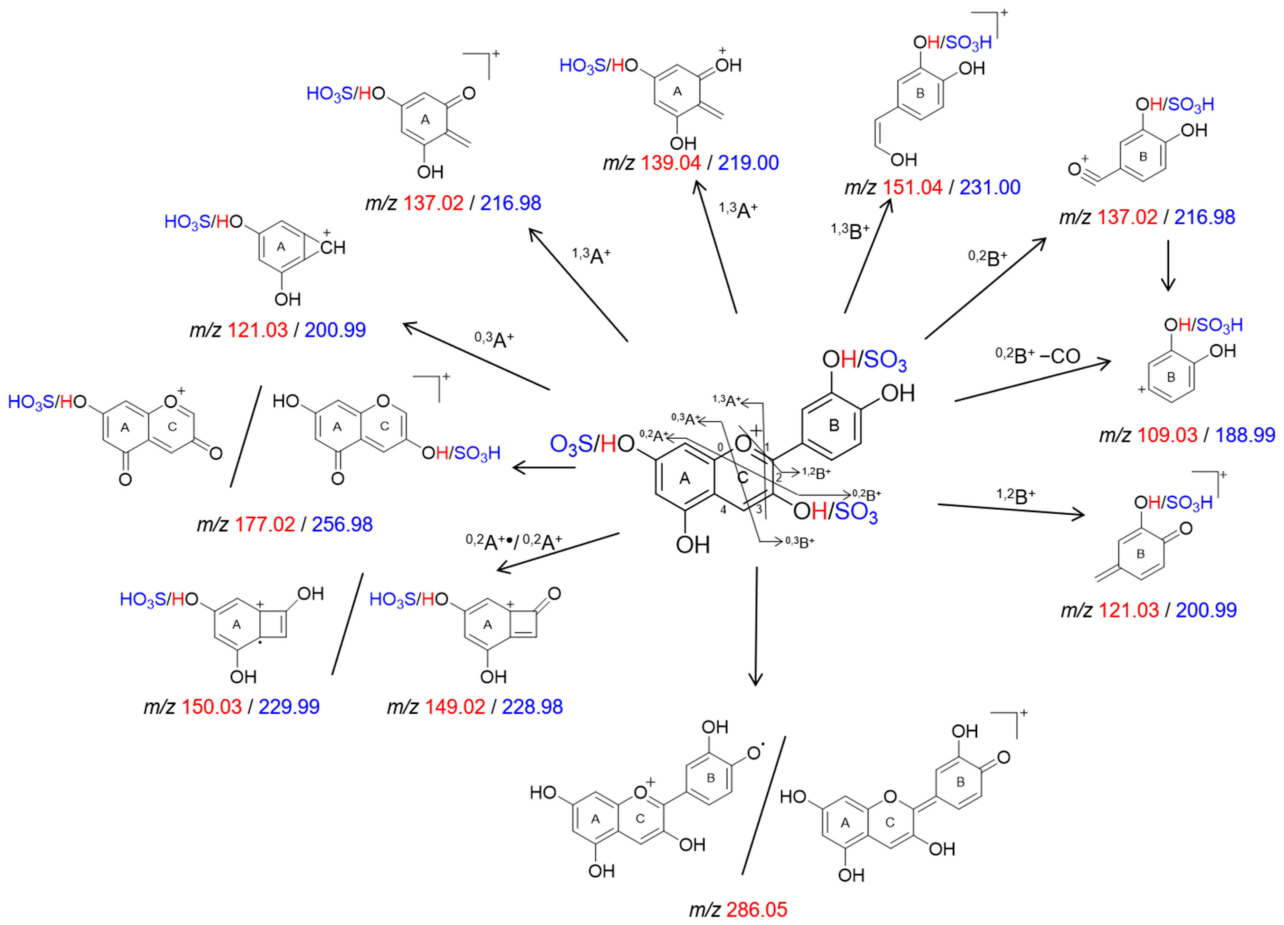
Postulated ESI^+^ fragmentation scheme for cyanidin (red) and its derivatives (blue) sulfated on the A, B or C ring, based on the proposals of Barnes and Schug [13], González-Manzano et al. [14], and Oliveira et al. [15]. Conjugation positions are arbitrarily selected and may occur at any OH group.

Mass spectrometry is one of the most valuable tools for the characterization of compounds and may even be superior to NMR spectroscopy when only small amounts are available. In the case of flavonoids, the small fragments emerging from the cleavage of the C ring are most valuable for identification purposes. The introduction of a sulfate group leads to a mass increment of 79.9569 u in the spectrum. These characteristic fragments and their corresponding sulfated forms are shown in Figure 3 and the masses observed via LIT-MS or TOF-MS analyses are listed in Table 1. Because the flavylium cation is very stable and the sulfate group represents a good leaving group leading to a neutral loss fragment, only limited fragmentation occurs and the fragments smaller than 286/287 u ([M − 80]^+^) are of little diagnostic benefit.

However, a tentative assignment of the individual peaks to a conjugation position on the A-, B- or C ring is possible. Peak **1** shows numerous small fragments. The fragment at *m*/*z* 149.02 originates from the cleavage of the A ring (^0,2^A^+^). Since it is not present in sulfated form, sulfation on the A ring can be excluded. Furthermore, fragments at *m*/*z* 121.03 (^0,3^A^+^ or ^1,2^B^+^) and 137.02 (^1,3^A^+^ or ^0,2^B^+^) were obtained, which may be both A- and B ring fragments. Because no fragment corresponding to the sulfated form was observed, it can be concluded that sulfation took place on the C ring. This assumption is confirmed by the simultaneous presence of a fragment at *m*/*z* 177.02 ([M − C_6_H_5_O_2_]^+^) and its sulfated form at *m*/*z* 256.98.

The spectra of peaks **2** and **3** look very similar, in here fragments at *m*/*z* 137.02 (^1,3^A^+^ or ^0,2^B^+^), 139.04 (^1,3^A^+^), and the respective sulfated forms at *m*/*z* 216.98 and 219.00 are detectable. In addition, fragments at *m*/*z* 177.02 ([M − C_6_H_5_O_2_]^+^) and 256.98 are present. Furthermore, the fragment at *m*/*z* 109.03 (^0,2^B^+^ − CO) but not its sulfated version can be found. These combinations lead to the conclusion that the A ring carries the sulfate group in these two compounds.

Peaks **4** and **5** do not show characteristic fragments, but also look very similar. However, applying the exclusion principle and considering their very similar retention times it can be concluded that these compounds correspond to cyanidin sulfated at the 3′-OH and 4′-OH groups of the B ring, respectively.

All peaks show a fragment at *m*/*z* 286.05, but with different intensities. Interestingly, the ratio of the fragment at *m*/*z* 286.05 to 287.02 is relatively constant and is 0.04 for peak **1**, 0.07 for peak **2**, and 0.01 for peak **3**. In peak **4**, this ratio reverses to 17 and the fragment at *m*/*z* 287.02 is hardly detectable. The last peak (**5**) shows both fragments, in some analyses with a ratio of 0.07 and in some with a ratio of 17. A possible explanation is that the fragment at *m*/*z* 286.05 belongs to one of the structures shown in Figure 3 ([M − 80]^+^ or [M − 80]^+^) and may be related to the radical scavenging properties of flavonoids [16]. The *o*-dihydroxy structure in the B ring confers high stability to a radical form and participates in electron delocalization [17].

It should be mentioned that some fragments were found only in the mass spectra recorded with the ion trap and some only in the spectra recorded with the TOF-MS. These differences may be due to the different collision gases used. The fragments are listed in Table 1 and therefore the differences are not explicitly mentioned in the descriptions of the spectra.

Another useful tool to distinguish compounds is ion mobility spectrometry and the resulting CCS values. If the measurements were taken on the same day, the values are very constant. In the case of the cyanidin sulfates, the CCS values are in the range of 180.8 Å^2^ for a probable B ring sulfate and 187.6 Å^2^ for a probable A ring sulfate. Thus, the distinction of the individual components can be accomplished based on these CCS values. 

Founded on the data discussed above, the elution sequence of the monosulfated compounds is C ring sulfate < A ring sulfate < B ring sulfate.

### 2.2. Cyanidin-3-O-Glucoside Sulfates

**Figure 4:**
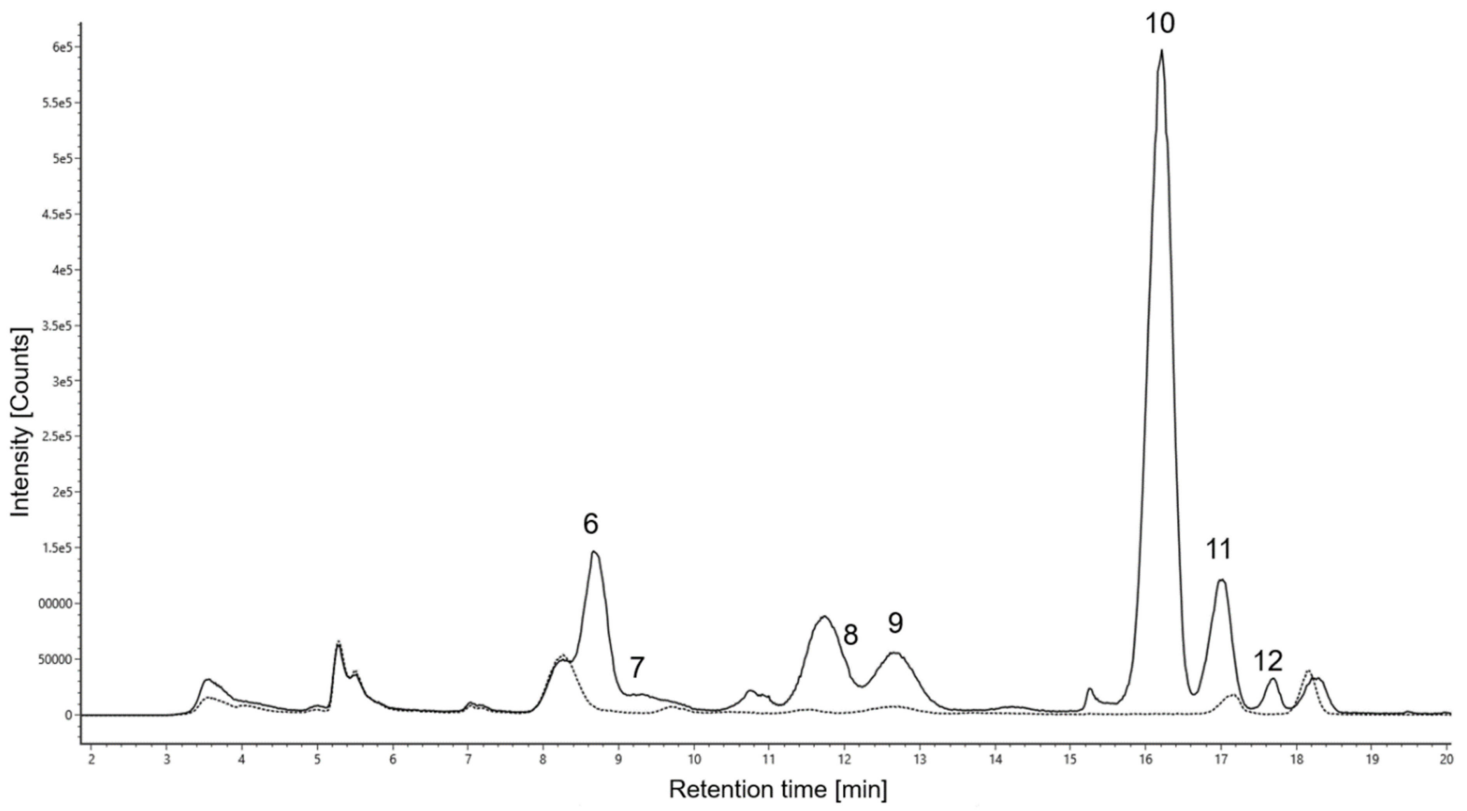
Extracted ion chromatograms of the [M]^+^, *m*/*z* 529.0646 (black line) and 609.0215 (dashed line) (cyanidin-3-*O*-glucoside mono- and disulfate) after the synthesis reaction.

In contrast to the cyanidin aglycone, there is no free OH group in the C ring that is available for sulfation. Therefore, conjugation of cyanidin-3-*O*-glucoside can take place only on the A- and the B rings. Figure 4 shows extracted ion chromatograms at *m*/*z* 529.06 and 609.02, corresponding to the masses of mono- and disulfated cyanidin-3-*O*-glucoside.

The yields determined at 280 nm were 4.4% for compound **6**, 3.0% for compound **8**, 3.8% for compound **9**, and 26.9% for compound **10**. All other monosulfated compounds were obtained at yields under 1%. Table 2 illustrates the data used for identification purposes, that is, retention times, accurate masses, CCS values, *m*/*z* of the most abundant fragments, relative yield, and absorption maxima.

#### 2.2.1. Retention Time

Four compounds (**6**, **7**, **8**, **9**) at *m*/*z* 529.06 elute earlier and three (**10**, **11**, **12**) later than cyanidin-3-*O*-glucoside. From this elution behavior it can be concluded that these two clusters differ in the site of conjugation. Furthermore, the first cluster can be divided into two groups. Peaks **6** and **7** elute at similar retention times as well as peaks **8** and **9**. Considering the elution order of the sulfated cyanidin aglycone and assuming that the sugar moiety does not influence the elution behavior, the two earlier eluting peaks can be allocated to A ring sulfates and the other two to B ring sulfates.

#### 2.2.2. UV Spectra

UV spectral data obtained for sulfation of cyanidin-3-*O*-glucoside were generally similar to those described for the aglycone. Because of the low concentrations, however, intensities were less pronounced. The absorptions at approximately 280 nm remained largely unchanged. Sulfation of compound **9** led to the largest shift of the λ_max_, that is, from 516 nm for cyanidin-3-*O*-glucoside to 493 nm. The additional band at about 420 nm already described for the cyanidin sulfates (peak **1** and **4**) was observed at 407 nm. Based on this data, the above-mentioned peak assignment is confirmed.

#### 2.2.3. Mass Spectrometry

The mass spectrometric data (Table 2) allowed the distinction of the above-mentioned two clusters of peaks. Compounds eluting earlier than 15 min showed a neutral loss of 162 u caused by the loss of the glucose moiety. These carry the sulfate group at the aglycone. In contrast, for molecules eluting later than 15 min, a neutral loss of 242 u was observed, which is indicative of a sulfate group at the sugar moiety. Unfortunately, an unambiguous assignment is not possible to a distinct position by means of mass spectrometry because the sulfate group is an excellent neutral loss fragment. However, the intensities of the resulting fragment signals (*m*/*z* 287.06 [M − 80 − 162]^+^, 367.01 [M − 162]^+^, and 449.11, [M−80]^+^) differ for each peak (Table 2). This behavior can be exploited, in addition to the retention time, for the identification of metabolites in physiological samples when their isolation is not possible, and references are not available. Because the four peaks eluting early (compounds **6**, **7**, **8** and **9**) could be allocated to a sulfated aglycone, it is evident that all hydroxyl groups are sulfated, yet some of them seem to be preferred. Whereas compound **6** was obtained at a yield of 4.5%, compound **7** was produced to such a small extent that no yield could be determined. This preference might be attributed to the differences in the pK_a_ value of the hydroxyl groups, which is the lowest for the 5-OH group [18]. In the case of the sugar moiety, steric reasons may be the explanation for differences in the site of sulfation [19]. Furthermore, higher temperatures during the reaction lead to an increased formation of disulfates, which is described in the literature [19] and which was also observed in this work.

Interestingly, we observed decreasing CCS values with increasing retention times within the monosulfates or disulfates, respectively. It may be concluded that smaller molecules, whose size can be derived from the shorter drift times, interact more with the column material, which results in longer retention times. This observation was also made with the metabolites that resulted from cyanidin and cyanidin-3-*O*-glucoside through liver enzymes [4].

### 2.3. Challenges

Some difficulties with the sulfation of small molecules are already described in the literature [6]. As inorganic salts may pose a problem, particularly in smaller approaches, they may cause inconsistencies concerning the purification and thus the yield. While the separation of quercetin sulfates by preparative HPLC has already been reported to pose a problem, [20] it may be even more difficult in the case of anthocyanins because of their pH-dependent structure. Additionally, the instability of the sulfate groups under acidic conditions and at high temperatures are pointed out in the literature [6]. The synthesis of resveratrol sulfates has even been referred to as a nightmare that can be handled only by selective protection of the OH groups [21]. Protective groups, which are often used for flavonoid derivatization, should be avoided for anthocyanins because their introduction and removal are often associated with unfavorable conditions. In contrast, the method applied in this study allows the production of sulfated derivatives without using protective groups. Another issue is the solvolysis of phenolic sulfates, which has been reported for 3- and 4-*O*-resveratrol sulfates during evaporation to dryness, probably caused by the presence of sulfuric acid as a reaction byproduct [22]. These and other challenges are the reasons why anthocyanins rarely appear in the long history of sulfation of phenolic substances [23]. Furthermore, with respect to the intensities, it should also be considered that the anthocyanin monosulfates may be converted to disulfates. Therefore, fewer monosulfates should be present at higher temperatures and longer reaction times.

Although LC-MS analysis is extremely useful for metabolite characterization, challenges still exist in the assignment of the peaks to the right number of sulfates. The retention times of some of the monosulfates are close to the disulfates and the disulfates produce monosulfates as in source fragments.

### 2.4. Human Pilot Study

In a first pilot study comprising only two individuals, we investigated urine samples for the presence of anthocyanin phase II metabolites after ingestion of a commercial blackberry nectar sample. The qualitative profile of the investigated metabolites in the two urine samples was comparable. Their analytical data such as the accurate mass, the main fragment, and the CCS values are given in Table 3. Among the metabolites detected, two sulfated cyanidin derivatives and one sulfated cyanidin-3-*O*-glucoside were found. The latter elutes after 13.1 min (gradient b), which corresponds to the retention time of the reference sulfate **9**. Their CCS values are also in the same range. However, it should be noted that while the deviations observed within a measurement are remarkably small, larger differences between different measurements may occur. For example, day-to-day variations in the CCS value for cyanidin of 162.5 ± 0.1 Å^2^ and on another day 165.5 ± 0.1 Å^2^ were observed, which results in an overall deviation of ± 2.1 Å^2^. Thus, the deviation of the CCS value of the synthesized sulfate **9** (216.2 ± 2.4 Å^2^) from the sulfate found in urine (209.4 ± 0.6 Å^2^) with nearly the same retention time can be justified.

The earliest peak corresponding to cyanidin sulfates that were detected in the urine samples elutes after 9.8 min (gradient a), which matches the retention time of peak **2**. The CCS value of this compound is 180.0 ± 1.0 Å^2^ and hence approximately 2 Å^2^ below that of the reference compound, which can be explained by the fact that measurements were not taken on the same day.

Taking into account these deviations, the compound eluting after 12.7 min can be assigned to peak **4** of the synthesized sulfates. The CCS value also of this peak **4** is approximately 2 Å^2^ higher than that of the substance detected in the urine.

Based on the mass spectrometric data, the compound eluting at 9.8 min can be assigned to an A ring sulfate, whereas the compound eluting after 12.7 min is sulfated at the B ring. To the best of our knowledge, this is the first work that reports a flavonoid metabolized to an A ring sulfate. Studies conducted on quercetin demonstrated the formation of quercetin-3′-*O*-sulfate in rat liver [7], which is a metabolite also present in humans [8]. From these findings, it may be deduced that compound **4** corresponds to cyanidin-3′-*O*-sulfate. As the 3′-*O*-sulfate was also obtained in the highest yields by other chemical syntheses [7], it may be concluded that the most intense peak in the synthesis of cyanidin sulfates is the 3′-*O*-sulfate. These results would confirm that the human body produces mainly 3′-*O*-sulfates of flavonoids [8].

## 3. Materials and Methods

### 3.1. Chemicals

**Scheme 2:**
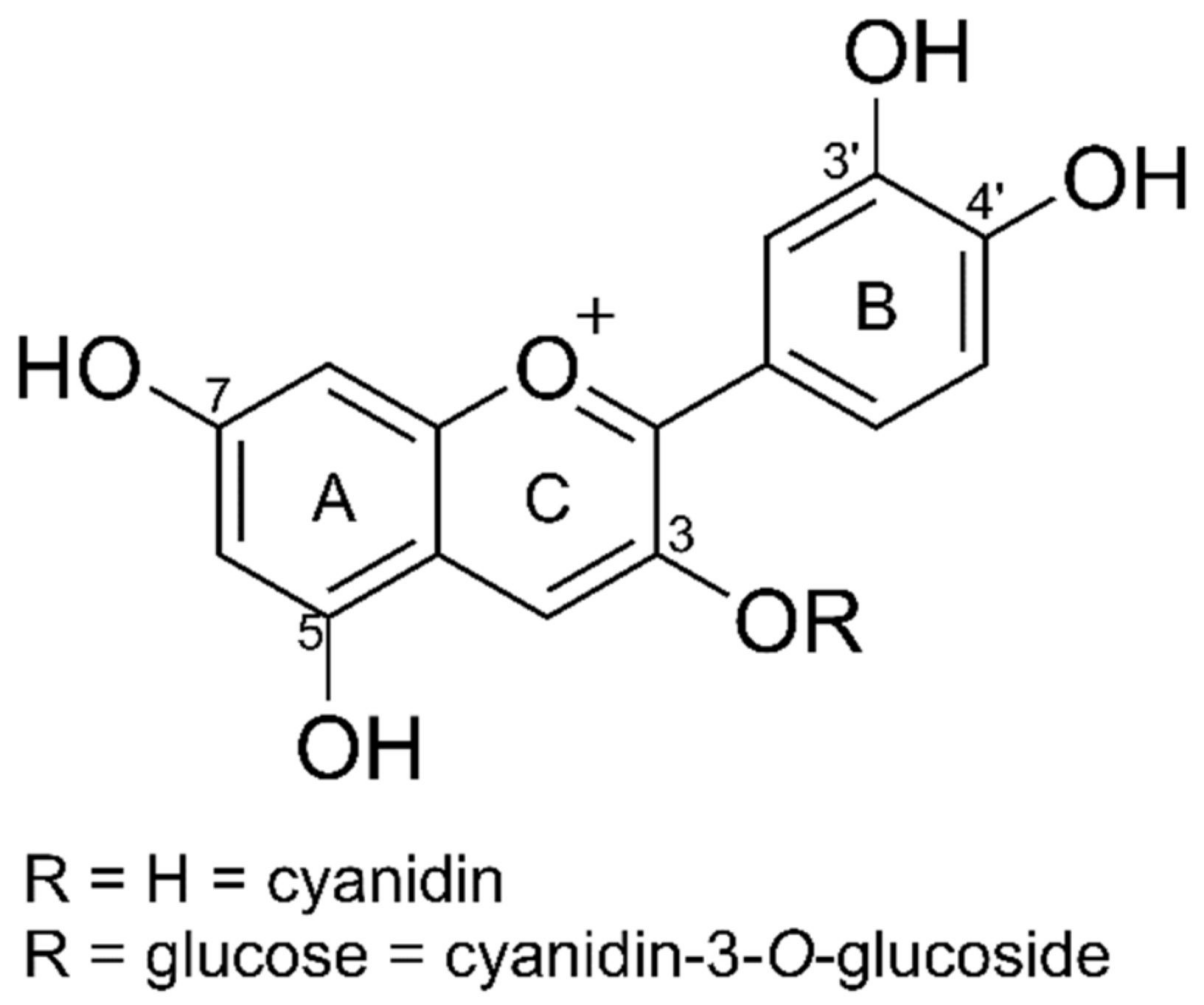
Structure of cyanidin and cyanidin-3-*O*-glucoside.

LC-MS-grade water, acetonitrile, formic acid, and ammonium hydroxide were purchased from ChemSolute (Renningen, Germany). Sulfur trioxide-*N*-triethylamine complex, leucine enkephalin acetate salt hydrate, and pyridine were obtained from Sigma-Aldrich (St. Louis, MO, USA). *N*,*N*-Dimethylformamide (DMF) was from Fisher Scientific (Waltham, MA, USA). Methanol, ascorbic acid, and hydrochloric acid (HCl) were supplied by VWR (Darmstadt, Germany). Trifluoroacetic acid (TFA) was purchased from Alfa Aesar (Ward Hill, MA, USA). An anthocyanin extract containing primarily cyanidin-3-*O*-glucoside (Scheme 2) was obtained as described before [4]. To obtain the cyanidin aglycone, the lyophilized extract was treated with concentrated HCl for 90 min at 90 °C.

### 3.2. Hemisynthesis of Sulfated Metabolites

The approach is based on the method described by Jones et al. [7] and the equations in Scheme 1 graphically illustrate the two reactions of cyanidin and cyanidin-3-*O*-glucoside, respectively. Cyanidin-3-*O*-glucoside (7 µmol) was dissolved in approximately 20 mL dry pyridine to remove any water that may be present. Pyridine was evaporated under reduced pressure and the entire procedure was repeated. The dried compound was dissolved in 1 mL DMF. The cyanidin aglycone (7 µmol) was dissolved directly in 1 mL DMF. This mixture was then transferred to an Eppendorf tube and allowed to react with a 10-fold excess of sulfur trioxide-*N*-triethylamine complex in a thermomixer (Eppendorf, Hamburg, Germany). A temperature of 50 °C and a reaction time of 120 min were found to be optimal for the hemisynthesis of monosulfated cyanidin-3-*O*-glucoside; for the cyanidin aglycone, optimum conditions were 45 °C and 60 min, respectively. These conditions allowed both maximizing the concentration of the monosulfates and minimizing the formation of multiple sulfated conjugates. After 5-fold dilution with water and acetonitrile containing 0.1% formic acid, and microfiltration (regenerated cellulose, 0.20 μm), the samples were analysed using LC-LIT-MS^n^ and LC-IMS-QTOF-MS.

The polarity of non-charged flavonoids is increased by sulfation in a way that they precipitate in the used solvent, which is mostly dioxane [7]. In the case of anthocyanins, the polarity of the resulting substances is similar to the parent molecules, so that they do not precipitate. Therefore, solid-phase extraction with a weak anion exchanger was applied to separate the cyanidin-3-*O*-glucoside sulfates from the starting material to take advantage of the different local charge distribution. For this purpose, OASIS WAX cartridges (6 cc Vac cartridge, 500 mg sorbent per cartridge, 60 µm particle size; Waters, Milford, MA, USA) were washed with methanol and water. After dilution of the samples with 4 mL water, aliquots of 2 mL were applied to the ion exchanger; subsequently, the material was washed again with water containing 2% of formic acid. Most of the unreacted cyanidin-3-*O*-glucoside was removed using 100% methanol in the first elution step. The sulfated target compounds were then eluted with 5% ammonia in methanol, 3% HCl in water/methanol 50:50 (*v*/*v*), and 20% trifluoracetic acid in water/methanol 50:50 (*v*/*v*) until the eluates were colorless (approx. 3–5 mL). Eluates were evaporated under a constant nitrogen stream to dryness and redissolved in 250 µL of acetonitrile/water/formic acid 20:79.9:0.1 (*v*/*v*/*v*).

### 3.3. LC-MS-Analysis

UHPLC analysis of the reaction products was performed on an Acquity UPLC I-Class system (Waters, Milford, MA, USA) consisting of a binary pump, a sample manager cooled at 10 °C, a column oven set at 40 °C, and a diode array detector scanning from 250 to 650 nm. An Acquity HSS-T3 RP18 column (150 mm × 2.1 mm; 1.8 µm particle size) combined with a pre-column (Acquity UPLC HSS T3 VanGuard, 100 Å, 2.1 mm × 5 mm, 1.8 µm), both from Waters (Milford, MA, USA) was used for separation with water (A) and acetonitrile (B) as eluents, both acidified with 0.1% (v + v) formic acid. The flow rate was set at 0.4 mL/min. Since the reaction products differ considerably in their polarity, two different gradients had to be applied to achieve the best separation possible. Sulfated reaction products of the cyanidin aglycone were analyzed using a linear gradient from 8% B to 10% B for 5 min, then to 17% B for 6 min, to 30% B for 4 min, and to 50% B for 8 min (gradient a). Sulfates of cyanidin-3-*O*-glucoside were analyzed using a gradient whose solvent composition changed from 1 to 10% B in 13 min, then to 17% B in 6 min, and then to 30% B in 4 min (gradient b). The injection volume was 5 µL. For MS analysis, the UHPLC was coupled with an LTQ-XL ion trap mass spectrometer (Thermo Scientific, Inc., Waltham, MA, USA) equipped with an electrospray interface operating in the positive ion mode. Ion mass spectra were recorded in the range of *m*/*z* 245–1000. The capillary was set at 325 °C with a spray voltage of 16 V for cyanidin and 30 V for cyanidin-3-*O*-glucoside. The source voltage was maintained at 4 kV at a current of 100 µA and the tube lens was adjusted to 55 V for cyanidin and 75 V for cyanidin-3-*O*-glucoside. Nitrogen was used as sheath, auxiliary and sweep gas at a flow of 70, 10 and 1 arb, respectively. Three consecutive scans were conducted with helium as collision gas: a full mass scan, an MS^2^ scan of the most abundant ion of the first scan using normalized collision energy (CE) of 20%, and an MS^3^ analysis of the most abundant ion in the MS^2^ experiment with a CE of 35%. In addition, multiple reaction monitoring (MRM) measurements were performed and the masses resulting from typical losses of one [M − 80]^+^ or two [M − 80 − 80]^+^ sulfate groups or glucose [M − 162]^+^ were scanned. Data were acquired and processed using Xcalibur 2.2SP1.48 (Thermo Scientific, Inc., Waltham, MA, USA).

For ion mobility spectrometry measurements, the UHPLC was connected to a Vion IMS QTOF mass spectrometer (Waters, Milford, MA, USA) operating in the positive ion mode. The capillary voltage was 0.5 kV, the source temperature was 120 °C, the cone voltage was 40 V, the desolvation gas temperature was 550 °C, and the desolvation gas flow was 1200 L/h. The measurements were conducted with automatic lock correction every 5 min with leucine enkephalin as the lock mass at a concentration of 100 pg/µL. Nitrogen was used as the drift and collision gas and the MS mode was high definition with a low collision energy of 6 eV and a high collision energy ramp of 20–40 eV. Data were acquired and processed using UNIFI v1.9.2.045 (Waters, Milford, MA, USA).

### 3.4. Study Design and Analysis of Urine

As a proof of concept, the urine of two people was collected over 24 h after consumption of 400 mL commercial blackberry nectar. This volume contained about 100 mg cyanidin-3-*O*-glucoside. Additionally, one sample of urine was taken prior to consumption.

The diet was anthocyanin-free in the week before the study and polyphenol-free on the day of the study. According to the procedure of Feliciano et al. [24], ascorbic acid was added to the urine containers (5.63 g/3 L container). Aliquots were adjusted to pH 2.5 with formic acid for stabilization and measured directly to avoid precipitation of the desired metabolites [25]. Direct measurement was required because we had observed that after one month of storage of the samples at −80 °C, the peak intensities were significantly lower.

### 3.5. Sample Preparation

Samples were prepared according to a previously published protocol [26]. Briefly, 5 mL of a urine sample was evaporated at 40 °C under nitrogen flow to a final volume of 500–700 µL. After microfiltration (regenerated cellulose, 0.20 µm), the samples were analysed using LC-IMS-QTOF-MS and LC-LIT-MS.

## 4. Conclusions

Our knowledge about the fate of anthocyanins in the human body is still incomplete. It is known that after absorption various enzymes transfer glucuronic acid and/or a sulfate group as well as a methyl group to the aglycone or glycoside. Additionally, cleavage of the glycoside and other reactions are possible. From the results of the human pilot study, it is evident that the metabolite species mentioned above can be found in various forms in urine. It has been possible to chemically synthesize sulfates of cyanidin and cyanidin-3-*O*-glucoside and to use these as reference substances. Thus, two different cyanidin sulfates and one cyanidin-3-*O*-glucoside sulfate could be identified. In addition, two glucuronides and four combinations of glucuronidated and methylated derivatives of cyanidin were detected in human urine. Despite the difficulties associated with the small quantities from chemical synthesis, the structural elucidation of the sulfated anthocyanins is an advance in the complete characterization of their metabolic profile.

## Figures and Tables

**Table 1:** LC-DAD-ESI-LIT and LC-DAD-ESI-IMS-QTOF results of the synthesis reaction of cyanidin sulfates.

Peak No.	RT ^a^ (min)	[M]^+^ Calculated (*m*/*z*)	[M]^+^ Measured (*m*/*z*)	Mass Error (mDa)	Characteristic Fragment Ions MS^2^ (*m*/*z*) QTOF	Characteristic Fragment Ions MS^2^ (*m*/*z*) LIT	Degree of Sulfation	CCS Value /[M]^+^ (Å^2^)	λ_max_ (nm)	Supposed Location of Sulfate Group
-	4.4	446.9686	446.9691	+0.5	286.0469, 367.0120	287, 367	2	198.9 ± 0.6	250, 385, 505	n.d.
-	4.8	446.9686	446.9684	−0.2	287.0550, 367.0117	287, 367	2	196.3 ± 0.1	n.d.	n.d.
-	5.5	446.9686	446.9692	+0.6	287.0552, 367.0120	287, 367	2	204.5 ± 0.6	278, 487	n.d.
-	6.5	449.1078	449.1079	+0.1	287.0551	287	*	204.6 ± 0.2	278, 516	
1	7.5	367.0118	367.0118	±0.0	121.0285, 137.0234, 149.0235, 177.0183, 256.9752, 286.0492, 287.0551	137, 177, 257, 287	1	183.6 ± 0.3	267, 410, 505	C ring
-	7.6	446.9686	446.9689	+0.3	287.0550, 367.0121	287, 367	2	203.7 ± 0.3	281, 505	n.d.
-	9.3	446.9686	446.9686	±0.0	286.0473, 367.0123	287, 367	2	202.7 ± 0.1	254, 372, 490	n.d.
2	9.9	367.0118	367.0118	±0.0	109.0284, 121.0285, 137.0235, 139.0390, 149.0238, 177.0185, 216.9805, 218.9785, 269.0447, 286.0475, 287.0551,	137, 139, 177, 217, 257, 269, 287	1	183.3 ± 0.1	275, 513	A ring
3	10.3	367.0118	367.0120	+0.2	121.0286, 137.0236, 139.0392, 149.0239, 177.0184, 219.0265, 256.9752, 287.0552,	137, 177, 257, 287	1	187.6 ± 0.1	281, 517	A ring
-	11.3	446.9686	446.9687	+0.1	286.0470, 367.0120	287, 367	2	189.6 ± 0.2	278, 329sh, 428, 497	n.d.
-	11.7	287.0550	287.0552	+0.2	109.0286, 121.0286, 137.0236, 149.0236, 269.0448	137, 149, 177, 269	cyanidin	163.4 ± 0.1	277, 443sh, 525	
4	12.6	367.0118	367.0118	±0.0	216.9807, 268.0370, 286.0471	137, 269, 287	1	182.6 ± 0.1	271, 429, 513	B ring
5	12.8	367.0118	367.0118	±0.0	268.0370, 269.0445, 286.0472, 287.0553	137, 269, 287	1	180.8 ± 0.2	283, 512	B ring

* Cyanidin-3-*O*-glucoside; n.d. = not determined; ^a^ = gradient a.

**Table 2:** LC-DAD-ESI-LIT and LC-DAD-ESI-IMS-QTOF results of the synthesis reaction of cyanidin-3-*O*-glucoside sulfates.

Peak No.	RT ^b^ (min)	[M]^+^ Calculated (*m*/*z*)	[M]^+^ Measured (*m*/*z*)	Mass Error (mDa)	Fragment Ions MS^2^ (*m*/*z*)	Degree of Sulfation	CCS Value [M]^+^ (Å^2^)	λ_max_
-	5.3	609.0215	609.0221	+0.6	529 (3.5), 447 (100), 367 (4.2)	2	240.8 ± 0.1	277, 505
-	5.5	609.0215	609.0219	+0.4	529 (5.1), 447 (100), 367 (5.4)	2	240.6 ± 0.1	278, 505
-	7.0	609.0215	609.0225	+1.0	529 (100), 447 (88.1), 367 (52.3), 287 (4.0)	2	237.5 ± 0.5	278
-	8.3	609.0215	609.0224	+0.9	529 (70.9), 367 (100), 287 (14.5)	2	232.3 ± 0.1	278, 500
6	8.8	529.0647	529.0650	+0.3	449 (2.8), 367 (100), 287 (5.3)	1	224.7 ± 0.1	278, 509
7	9.3	529.0647	529.0649	+0.2	449 (96.5), 367 (3.0), 287 (100)	1	217.1 ± 0.1	277, 504
-	9.8	609.0215	609.0223	+0.8	529 (100), 447 (2.3), 367 (18.7), 287 (3.3)	2	239.9 ± 0.2	278, 509
-	11.5	609.0215	609.0222	+0.7	529 (57.4), 447 (1.9), 367 (100), 287 (27.4)	2	236.2 ± 0.1	278, 492
8	12.0	529.0647	529.0652	+0.5	449 (7.5), 367 (100), 287 (4.5)	1	219.2 ± 0.1	278, 511
9	12.8	529.0647	529.0650	+0.3	449 (100), 367 (80.2), 287 (92.6)	1	216.2 ± 2.4	277, 407, 493
-	15.2	449.1078	449.1079	+0.1	287 (100)	*	204.6 ± 0.2	279, 516
10	16.4	529.0647	529.0651	+0.4	449 (100), 287 (99.6)	1	215.7 ± 0.2	279, 516
11	17.1	529.0647	529.0651	+0.4	449 (100), 287 (35.2)	1	218.2 ± 0.1	280, 516
-	17.2	609.0215	609.0224	+0.9	529 (100), 449 (2.3), 367 (1.8), 287 (19.3)	2	224.7 ± 0.2	280, 516
12	17.7	529.0647	529.0648	+0.1	449 (17.2), 287 (100)	1	214.9 ± 0.1	280, 516
-	18.2	609.0215	609.0220	+0.5	529 (100), 449 (21.8), 367 (1.1), 287 (3.9)	2	224.4 ± 0.1	280, 517

* cyanidin-3-*O*-glucoside; ^b^ = gradient b.

**Table 3:** LC-DAD-ESI-IMS-QTOF results of the analysis of urine collected for 24 h after the consumption of blackberry juice, either gradient a or b.

Suspected Compound	RT (min)	[M]^+^ Calculated (*m*/*z*)	[M]^+^ Measured (*m*/*z*)	Mass Error (mDa)	Fragment MS^2^ (*m*/*z*)	CCS Value (Å^2^)
**Cyanidin**						
unconjugated *	11.7 ^a^	287.0550	287.0549	−0.1		162.5 ± 0.1
glucuronidated	8.3 ^a^	463.0877	463.0871	−0.6	287	200.1 ± 0.9
	9.6 ^a^	463.0877	463.0874	−0.3	287	204.7 ± 1.0
glucuronidated and methylated	8.8 ^a^	477.1033	477.1029	−0.4	301	209.4 ± 2.4
	9.1 ^a^	477.1033	477.1030	−0.3	301	207.6 ± 0.7
	9.6 ^a^	477.1033	477.1028	−0.5	301	208.2 ± 0.7
	10.2 ^a^	477.1033	477.1029	−0.4	301	207.6 ± 0.4
sulfated	9.8 ^a^	367.0118	367.0118	±0.0	287	180.0 ± 1.0
	12.7 ^a^	367.0118	367.0118	±0.0	287	180.6 ± 0.5
**Cyanidin-3-*O*-glucoside**						
unconjugated *	15.0 ^b^	449.1078	449.1081	+0.3	287	203.3 ± 2.5
sulfated	13.1 ^b^	529.0647	529.0648	+0.1	287	209.4 ± 0.6

^a^ = gradient a; ^b^ = gradient b; * Not found in the samples, listed only for comparison.

## Data Availability

Data is contained within the article.

## Sample Availability

Samples of the compounds are not available from the authors.
